# A Comparison of Nest Structure and Materials in Three Species of Penduline Tits (*Remiz* spp.)

**DOI:** 10.1002/ece3.73935

**Published:** 2026-06-30

**Authors:** Zhe Hao, Yu Huang, Jiajun Jiang, Zhuoya Zhou, Wenni Jiang, Bengao Tang, Kuerbanjiang Hanahati, Kedeerhan Bayaheng, Zhengwang Zhang, Hui Wang, De Chen

**Affiliations:** ^1^ MOE Key Laboratory for Biodiversity Science and Ecological Engineering College of Life Sciences, Beijing Normal University Beijing China; ^2^ Guangdong Urban‐Rural Planning and Design Research Institute Technology Group Co., Ltd. Guangzhou China; ^3^ Xinyuan Forestry and Grassland Administration Xinyuan China; ^4^ Research Institute of Forestry Ili Kazakh Autonomous Prefecture Yining China; ^5^ Wildlife Protection and Management Center Bozhou Forestry and Grassland Administration Bole China

**Keywords:** beak morphology, body size, nest architecture, nest materials, penduline tits

## Abstract

Bird nest construction is shaped by both avian morphology and ecological context. In this study, we provide the first quantitative descriptions of nest structural parameters and material composition for the White‐crowned Penduline Tit ( *Remiz coronatus* ) and the Black‐headed Penduline Tit ( *R. macronyx* ). We also collected nest data for the Chinese Penduline Tit ( *R. consobrinus* ), allowing a descriptive comparison of nest architecture and nest materials across penduline tits. By quantifying nest structural parameters, nest mass, and material composition, we documented interspecific and interpopulation variation in several nest traits, including nest width, wall thickness, and corridor length. In the sampled nests, Chinese and White‐crowned Penduline Tits used mainly shredded plant stems, plant down, and wool, with mud additionally recorded in the White‐crowned Penduline Tit, whereas Black‐headed Penduline Tit nests consisted primarily of shredded reed leaves and reed down. Exploratory comparisons further suggested that populations with greater mean body length tended to have larger nest inner diameters, and that species with larger mean beak size tended to use wider nest fibers. Although these comparisons are descriptive only, our study provides new comparative natural history data on nest architecture and material use in penduline tits, laying a foundation for future work on the morphological and ecological drivers of nest construction in this group.

## Graphical abstract

We present the first quantitative descriptions of nest structural parameters and material composition for the White‐crowned and Black‐headed Penduline Tits. We further conducted a comparative analysis of nest architecture and materials in three penduline tit species ( *Remiz consobrinus* , *R. coronatus* , and *R. macronyx* ) across five sites in China by quantifying nest structure, mass, and material composition, and relating these traits to body length and beak size.

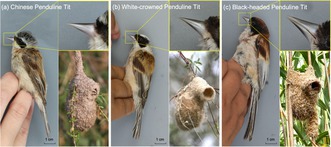

## 1 Introduction

Bird nests play a fundamental role in reproduction (Healy et al. 2023) by providing protection for eggs and nestlings against predators (Hung et al. 2022; Husby and Slagsvold 2025), parasites (Mainwaring et al. 2014), and adverse environmental conditions (Perez et al. 2020), while also buffering microclimatic variation during development (Sonnenberg et al. 2020; Thiruvenggadam et al. 2022; Corimanya et al. 2024). Nest structure (Street et al. 2022), size (Vanadzina et al. 2023), and material composition (Breen et al. 2021) can directly influence incubation efficiency (Akresh et al. 2017), nestling growth (Moreno et al. 2010), and ultimately reproductive success (Medina et al. 2022; Deeming 2023; Chou et al. 2025). As a result, nest construction is widely regarded as an important component of avian life‐history strategies (Healy et al. 2023) and a key interface (Perez et al. 2020) between birds and their environments.

Birds experience diverse environmental pressures across their breeding ranges, including variation in temperature, precipitation, vegetation structure, and substrate availability. In response, many species exhibit flexible nest‐building strategies (Perez et al. 2023), adjusting nest size (Mazgajski et al. 2025), architecture (Perez et al. 2020), and material choice (Sugasawa et al. 2025) to local conditions in order to enhance reproductive performance (Dickinson et al. 2022; Perez et al. 2023; Sheard et al. 2024). Such variation may arise not only from environmental constraints but also from intrinsic morphological traits, such as body size and beak morphology (Healy et al. 2023; Sheard et al. 2023; Akresh et al. 2024; Li et al. 2024), which determine the physical capacity to manipulate nesting materials and construct nests of particular sizes and forms. Considering the interplay between morphology, nest structure, and material use can therefore provide useful insights into the ecological and evolutionary context of nest‐building behavior.

Penduline tits (genus *Remiz*), comprising four extant species (Wang et al. 2026; AviList Core Team 2025), are widely recognized for their distinctive nest architecture. The Eurasian Penduline Tit ( *Remiz pendulinus* ), White‐crowned Penduline Tit ( *R. coronatus* ), and Chinese Penduline Tit ( *R. consobrinus* ) generally construct pendulous nests suspended from the upper branches of trees, whereas the Black‐headed Penduline Tit ( *R. macronyx* ) builds its nest between two standing reed stems (Wang et al. 2023). Despite the iconic nature of these nests, detailed quantitative descriptions of nest structure and material composition are available primarily for the Eurasian Penduline Tit (Hoi et al. 1996; Zheng et al. 2018), and information on the Black‐headed Penduline Tit is particularly sparse. This uneven documentation, together with interspecific differences in habitat use and nesting substrates, highlights the value of a comparative description of nest structure and materials across the genus.

**FIGURE 1:**
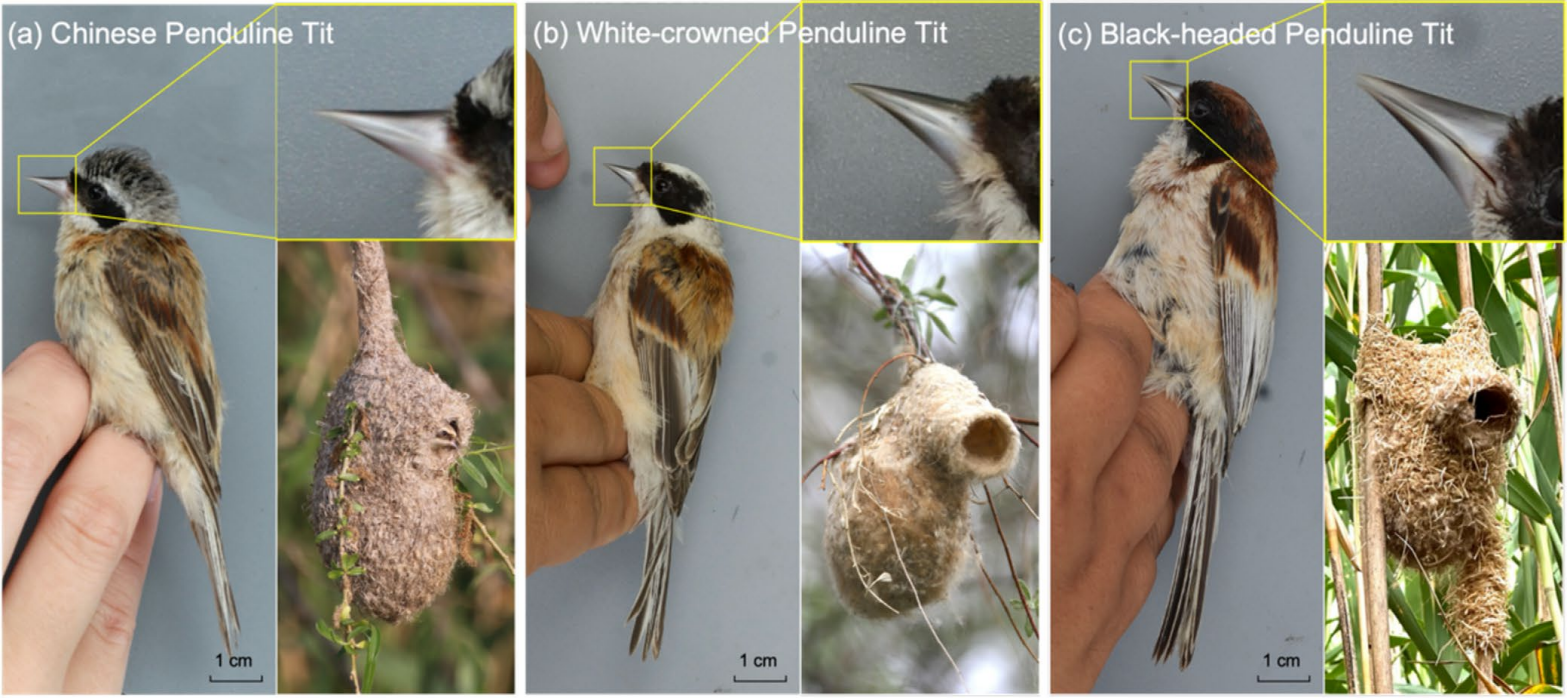
The three penduline tit species examined in this study. (a) Chinese Penduline Tit, including a close‐up of the beak and its nest; (b) White‐crowned Penduline Tit, including a close‐up of the beak and its nest; (c) Black‐headed Penduline Tit, including a close‐up of the beak and its nest.

In this study, we describe nest structure and material composition in three species of penduline tits, including the White‐crowned and Black‐headed Penduline Tits (Figure 1), for which quantitative nest measurements have not previously been reported. We also present descriptive comparisons of nest traits among populations and species. Given previous work suggesting that nest size may covary with body size (Vanadzina et al. 2023), we visually compared mean body length with mean nest inner diameter at the population level. We also compared mean beak size with mean nest fiber width at the species level because beak morphology may affect how birds manipulate and select nesting materials (Sheard et al. 2023).

## 2 Materials and Methods

### 2.1 Study Area

**FIGURE 2:**
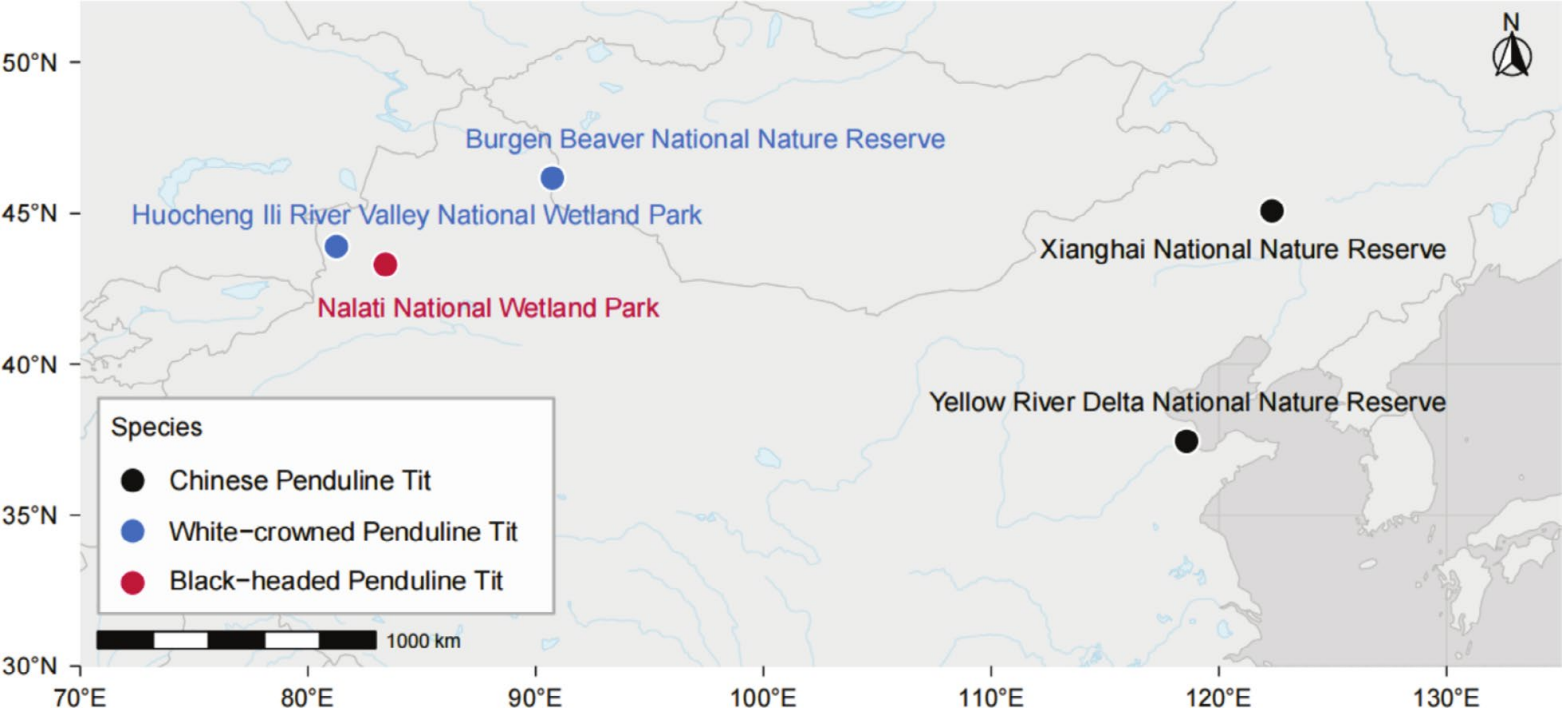
Sampling locations of the three penduline tit species. Black: Chinese Penduline Tit: Yellow River Delta National Nature Reserve and Xianghai National Nature Reserve; Blue: White‐crowned Penduline Tit: Burgen Beaver National Nature Reserve and Huocheng Ili River Valley National Wetland Park; Red: Black‐headed Penduline Tit: Nalati National Wetland Park. Basemap data were obtained from OpenStreetMap under the Open Data Commons Open Database License (ODbL), and the basemap style was adapted from CartoDB tiles licensed under CC BY‐SA 2.0.

We collected data on three species of penduline tits across five study sites in China. Chinese Penduline Tits were sampled at two locations: the Yellow River Delta National Nature Reserve (YRD) in Dongying City, Shandong Province (118°41′–119°16′E, 37°40′–38°10′N), and the Xianghai National Nature Reserve (XH) in Baicheng City, Jilin Province (122°05′–122°31′E, 44°55′–45°09′N). White‐crowned Penduline Tits were sampled at two sites in Xinjiang: Burgen Beaver National Nature Reserve (BG), Altay Prefecture (90°27′–91°00′E, 46°05′–46°15′N), and Huocheng Ili River Valley National Wetland Park (HC), Ili Prefecture (80°38′–80°40′E, 43°48′–43°50′N). Black‐headed Penduline Tits were sampled at Nalati National Wetland Park (NLT), Xinyuan County, Ili Prefecture, Xinjiang (82°28′–83°30′E, 43°03′–43°40′N) (Figure 2).

### 2.2 Bird Morphological Data

During the breeding season, penduline tits were captured using mist nets. For each individual, we measured body length, wing length, tail length, tarsus length, and beak morphology, including beak length, beak depth, and beak width. Body mass was measured using an electronic balance (±0.01 g), and linear measurements were taken with a ruler or vernier caliper (±0.01 mm). After measurement, birds were fitted with color rings and released once their condition had been confirmed to be normal. All capture and handling procedures were conducted under the required permits and ethical approval.

Morphological data were collected during the breeding season between 2020 and 2025, but not all sites were sampled in the same year. To reduce measurement error across sites and years, all observers received standardized training before data collection and followed the same measurement protocol throughout the study. Trait definitions and measurement positions were standardized in advance, and the same instruments were used across sites whenever possible.

### 2.3 Nest Data

#### 2.3.1 Nest Collection

During the breeding season, we systematically searched for nests at the five study sites. Nest ownership was determined through observations of color‐ringed individuals, and nest stage was recorded through regular nest checks. Based on changes in nest morphology, penduline tit nests can be classified into stages A–F (Zheng et al. 2018). After the breeding season, a subset of intact stage F nests was randomly collected and brought to the laboratory. All nests were collected only after breeding had finished, and all removed nests were reattached near their original sites before the following breeding season.

To minimize pseudoreplication, we defined the sampling unit at the level of the individual nest. Nest ownership was assigned from observations of color‐ringed individuals and long‐term banding records, which confirmed that no nest included in the analyses was built by the same breeding pair. Nests were also spatially dispersed within each study area. At the Huocheng Ili River Valley National Wetland Park, which yielded the largest sample, breeding territories were typically separated by ~50 m, although neighboring nests occasionally occurred 20–30 m apart later in the season. In the Black‐headed Penduline Tit population at Nalati National Wetland Park, sampled nests were distributed along the wetland margin, with a minimum inter‐nest distance of ~70 m. Given the absence of repeated nests from the same pair and the broad spatial dispersion of nests within sites, we treated each completed (F‐stage) nest as an independent sampling unit.

#### 2.3.2 Nest Structure Parameters

**FIGURE 3:**
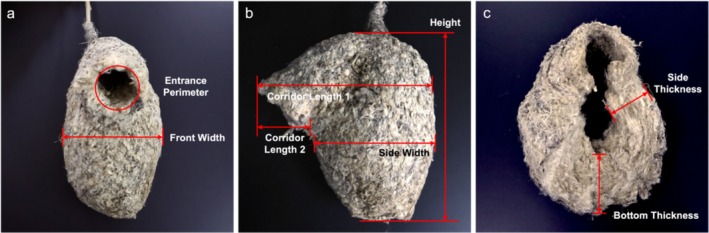
Schematic diagram of nest parameter measurements. (a) front view of the nest; (b) side view of the nest; (c) front sectional view of the nest. Front width and side width represent the maximum nest widths in frontal and lateral view, respectively. Height represents the maximum vertical length of the nest. Corridor length 1 was measured from the entrance edge to the opposite inner wall, and corridor length 2 from the entrance edge to the distal end of the corridor.

Nest structure was quantified using a standardized measurement protocol (Figure 3). Front width and side width were defined as the maximum widths in frontal and lateral view, respectively. Height was measured as the maximum vertical length of the nest. Corridor length 1 was measured from the entrance edge to the opposite inner wall, and corridor length 2 from the entrance edge to the distal end of the corridor. Nest height, frontal width, lateral width, corridor length 1, corridor length 2, and entrance perimeter were measured using a straight ruler (±0.01 mm). Bottom thickness and side thickness were measured using a vernier caliper (±0.01 mm). Nest inner diameter was calculated by subtracting twice the side thickness from the frontal width. This calculation was intended to estimate the effective internal space available for eggs and nestlings.

Nest volume was determined by completely filling each nest with fine sand and subsequently transferring the sand into a 1‐L graduated cylinder to obtain volumetric measurements.

To assess measurement consistency, we re‐measured a subset of 10 nests for bottom thickness, side thickness, and corridor length using the same standardized protocol and reference points. Repeated measurements showed no evidence of systematic bias between rounds and moderate to very high repeatability across traits, with particularly high consistency for bottom thickness. All nest traits were otherwise measured following fixed definitions (Figure 3), and uncertain measurements were re‐checked before being recorded.

#### 2.3.3 Nest Mass and Material Composition

For nests collected in 2025, 10 nests each of Chinese Penduline Tit and White‐crowned Penduline tit, and 7 nests from Black Headed Penduline tit, were selected for additional measurements. Before weighing, branches or reed stems attached to the nests were removed with scissors, and loose foreign material inside the nest chamber, including feathers, feces, and other debris, was carefully removed. Nests were then dried at 80°C for 24 h (Glądalski et al. 2025). After cooling to room temperature, the dry mass of each nest was measured using a precision balance (readability: 0.00001 g) and recorded to 0.01 g.

Nests of penduline tits are structurally composed of three distinct components: the structural layer, the lining, and the attachment (Hansell 2000), with the structural layer responsible for maintaining nest shape and integrity to prevent deformation or collapse, the lining being an independent inner layer that does not provide structural support, and the attachment serving to secure the nest to its substrate. Because the structural layer is relatively uniform within individual nests, and because fine‐scale separation of materials is particularly difficult in the wool‐rich nests of the White‐crowned Penduline Tit, we used a standardized subsampling approach. For each species, three nests were randomly selected, and from each nest three 1 × 1 cm samples were taken from the structural layer: the nest bottom, the area adjacent to the entrance corridor, and the side opposite the entrance corridor. Different nest material types were manually separated, weighed, and used to calculate the proportional composition of materials across species. Additionally, during the nest disassembly process, the types of nesting materials used by the three species of penduline tits were documented.

Besides, ten plant fibers (e.g., shredded bark strips and reed leaves) were sampled from the structural layer of each nest using forceps. The nest material fibers were then photographed under a ZEISS V8 stereomicroscope, and fiber diameters were subsequently measured.

### 2.4 Descriptive Comparison of Morphology and Nest Traits

As part of this descriptive study, we plotted mean ± SD values of selected morphological and nest traits for visual comparison. At the population level, we plotted body length against nest inner diameter for the sampled populations. The XH population of the Chinese Penduline Tit was excluded because side‐thickness data were unavailable, preventing estimation of nest inner diameter.

At the species level, we plotted beak size against nest fiber width for visual comparison among the three species. Because nest fiber width was measured only for nests collected in 2025, and no fiber‐width data were available for the BG and XH populations, this part of the study was limited to a species‐level comparison. Beak size was summarized using the first principal component (PC1) derived from standardized beak length, beak depth, and beak width measurements (Grant and Grant 2002; Sheard et al. 2023). No statistical analysis was performed.

## 3 Results

### 3.1 Bird Morphological Data

For the exploratory comparisons, we used morphometric data from 30 individuals from each of the five sampled populations (Table 1), giving a total of 150 individuals across the three penduline tit species.

**TABLE 1:** Morphometric data of three *Remiz* species (mm; mean ± SD).

Species	Wing length	Tail length	Body length	Tarsus length	Beak length	Beak depth	Beak width
Chinese Penduline Tit–YRD (*n* = 30; 2025)	53.69 ± 13.07	45.15 ± 1.30	112.72 ± 3.25	13.81 ± 0.43	9.59 ± 0.33	3.82 ± 0.16	3.69 ± 0.19
Chinese Penduline Tit–XH (*n* = 30; 2020)	55.19 ± 1.49	44.18 ± 2.01	100.82 ± 3.13	14.31 ± 1.05	9.32 ± 0.64	4.20 ± 0.21	—
White‐crowned Penduline Tit–HC (*n* = 30; 2025)	50.78 ± 1.28	42.73 ± 1.70	104.43 ± 2.27	13.00 ± 0.45	8.53 ± 0.56	3.60 ± 0.12	3.70 ± 0.17
White‐crowned Penduline Tit–BG (*n* = 30; 2023)	54.22 ± 1.22	44.62 ± 1.30	106.43 ± 2.18	13.46 ± 0.37	8.39 ± 0.32	3.60 ± 0.12	3.43 ± 0.22
Black‐headed Penduline Tit–NLT (*n* = 30; 2024)	60.00 ± 1.68	55.23 ± 1.89	125.65 ± 2.62	15.28 ± 0.37	10.43 ± 0.36	4.58 ± 0.19	4.15 ± 0.20

*Note:* “*n*” indicates sample size, and the year in parentheses indicates sampling year. “—” indicates missing data.

Abbreviations: BG, Burgen Beaver National Nature Reserve; HC, Huocheng Ili River Valley National Wetland Park; NLT, Nalati National Wetland Park. XH, Xianghai National Nature Reserve; YRD, Yellow River Delta National Nature Reserve.

### 3.2 Nest Structure Parameters

The final nest dataset included 64 nests of Chinese Penduline Tit, 159 nests of White‐crowned Penduline Tit, and seven nests of Black‐headed Penduline Tit (Table 2). Nest structural parameters varied among species and populations. Among the measured traits, inner diameter, corridor lengths, and entrance perimeter differed markedly among species. Black‐headed Penduline Tit nests generally had a larger entrance perimeter and inner diameter, but a shorter corridor than nests of the other two species. Nest wall thickness also varied among species. Bottom and side thickness were greatest in the Chinese Penduline Tit and smallest in the Black‐headed Penduline Tit. Nest volume ranged from 200.64 ± 42.24 to 256.07 ± 43.19 mL; the largest mean nest volume was observed in the White‐crowned Penduline Tit, whereas the smallest was recorded in the Chinese Penduline Tit.

**TABLE 2:** Nest structural parameters of three *Remiz* species (mm; mean ± SD).

	Chinese Penduline Tit		White‐crowned Penduline Tit		Black‐headed Penduline Tit
	YRD (*n* = 10; 2025)	XH (*n* = 54; 2019–2021)	HC (*n* = 145; 2024–2025)	BG (*n* = 14; 2024)	NLT (*n* = 7; 2025)
Height	147.40 ± 10.13	149.65 ± 17.40	140.20 ± 10.31	140.14 ± 11.67	151.43 ± 12.20
Front width	83.85 ± 6.71	85.77 ± 9.28	77.12 ± 6.84	89.64 ± 5.96	89.57 ± 9.29
Side width	83.35 ± 7.30	92.75 ± 9.53	79.07 ± 6.59	89.93 ± 5.06	84.29 ± 4.64
Inner diameter	46.05 ± 11.69	—	44.81 ± 8.96	52.06 ± 13.54	64.16 ± 6.11
Corridor length 1	120.50 ± 35.29	—	117.69 ± 15.67	116.93 ± 13.97	86.86 ± 16.72
Corridor length 2	38.45 ± 3.53	—	40.26 ± 13.01	36.29 ± 14.65	9.14 ± 3.55
Entrance perimeter	116.20 ± 13.02	—	103.59 ± 11.70	123.00 ± 12.57	129.43 ± 18.64
Bottom thickness	46.87 ± 13.57	66.65 ± 14.47	33.01 ± 9.71	30.86 ± 11.57	29.20 ± 8.56
Side thickness	18.90 ± 5.17	—	16.16 ± 4.34	18.79 ± 6.50	10.06 ± 2.12
Volume (mL)	213.90 ± 21.05	200.64 ± 42.24	225.25 ± 33.50	256.07 ± 43.19	203.57 ± 25.77

*Note:* “*n*” indicates sample size, and the year in parentheses indicates sampling year. A dash “—” indicates missing data.

Abbreviations: BG, Burgen Beaver National Nature Reserve; HC, Huocheng Ili River Valley National Wetland Park; NLT, Nalati National Wetland Park; XH, Xianghai National Nature Reserve; YRD, Yellow River Delta National Nature Reserve.

### 3.3 Nest Mass and Material Composition

This limited subsample provides a preliminary characterization of nest mass among the three penduline tit species. Nests of the Chinese Penduline Tit were heaviest on average (31.07 ± 6.65 g, *n* = 10), followed by those of the Black‐headed Penduline Tit (26.49 ± 4.37 g, *n* = 7), whereas nests of the White‐crowned Penduline Tit were lightest on average (23.45 ± 5.60 g, *n* = 10).

Based on the 2025 material subsamples (*n* = 3 nests per species), shredded plant stems and plant down constituted most of the measured nest material in all three species, although their relative proportions varied among species (Table 3). In the Chinese Penduline Tit, the measured material was composed mainly of plant down (61.85% ± 13.54%) and shredded plant stems (37.59% ± 13.34%), with only trace amounts of wool (0.56% ± 0.97%). In the White‐crowned Penduline Tit, the measured material included plant down (48.85% ± 2.05%), shredded plant stems (15.50% ± 12.84%), and wool (29.33% ± 18.82%); in addition, a small amount of mud was detected among different material layers at the nest base (6.32% ± 4.60%). In the Black‐headed Penduline Tit, the measured material consisted of shredded plant stems (48.97% ± 3.23%) and plant down (51.03% ± 3.23%), with no wool or mud detected in the sampled structural layer.

**TABLE 3:** Composition of nest materials used by the three *Remiz* species (%; mean ± SD).

	Chinese Penduline Tit (*n* = 3)	White‐crowned Penduline Tit (*n* = 3)	Black‐headed Penduline Tit (*n* = 3)
Shredded plant stems	37.59 ± 13.34	15.50 ± 12.84	48.97 ± 3.23
Plant down	61.85 ± 13.54	48.85 ± 2.05	51.03 ± 3.23
Wool	0.56 ± 0.97	29.33 ± 18.82	—
Mud	—	6.32 ± 4.60	—

*Note:* “—” indicates that the material was not detected in the sampled nests.

### 3.4 Width of Nest Material Fibers

**FIGURE 4:**
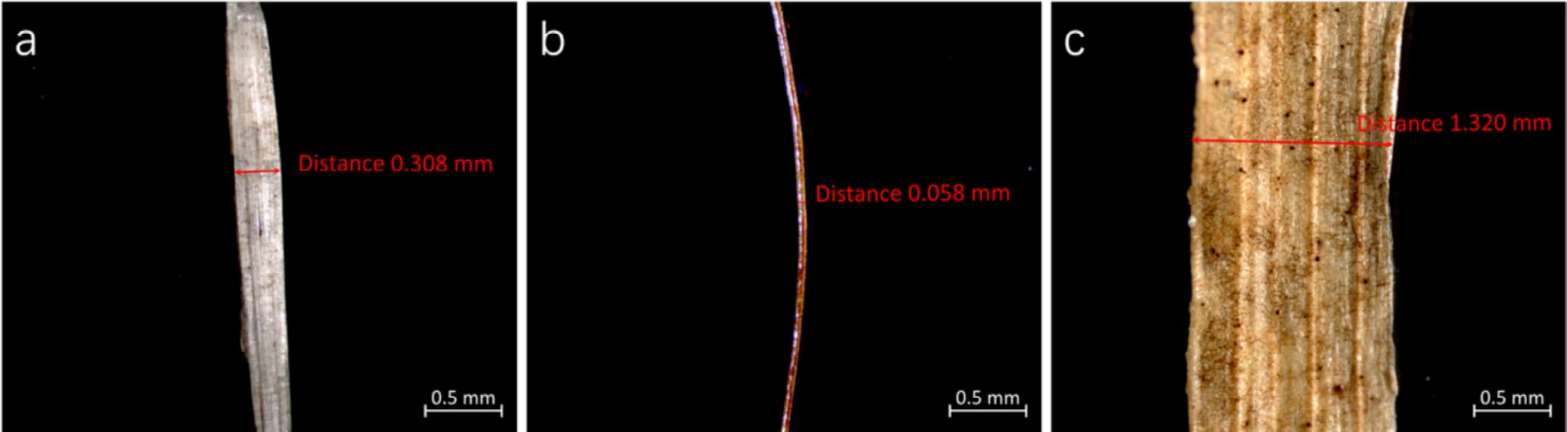
Nest material fibers of nests of three Remiz species collected in 2025. (a) nest material plant fibers of the Chinese Penduline Tit. (b) nest material plant fibers of the White‐crowned Penduline Tit. (c) nest material plant fibers of the Black‐headed Penduline Tit.

The widths of shredded plant fibers used in the nest structural layer differed among the three penduline tit species (Figure 4). Chinese Penduline Tit nests had a mean fiber width of 0.29 ± 0.03 mm (*n* = 10), whereas White‐crowned Penduline Tit nests had the narrowest fibers, with a mean width of 0.12 ± 0.04 mm (*n* = 10). Black‐headed Penduline Tit nests contained the widest fibers, with a mean width of 1.37 ± 0.18 mm (*n* = 10).

### 3.5 Descriptive Comparison of Morphology and Nest Traits

**FIGURE 5:**
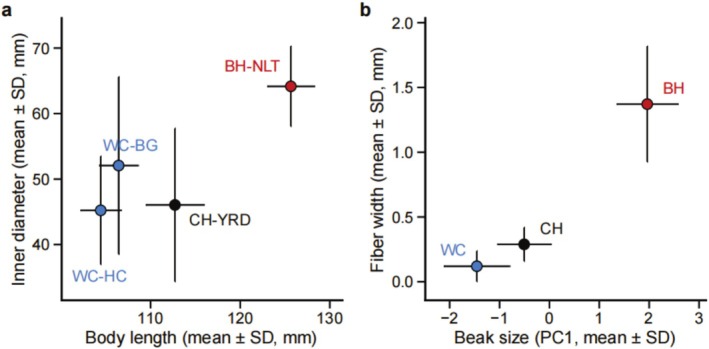
Relationships between morphological traits and nest structural characteristics. BH, The Black‐headed Penduline Tit; CH, The Chinese Penduline Tit; WC, The White‐crowned Penduline Tit. (a) Relationship between body length and inner nest diameters. BG, The Burgen Beaver National Nature Reserve; HC, The Huocheng Ili River Valley National Wetland Park; NLT, The Nalati National Wetland Park; YRD, The Yellow River Delta National Nature Reserve. (b) Relationship between beak size (PC1) and nest fiber width.

Figure 5a shows population means (± SD) of body length and nest inner diameter. The Black‐headed Penduline Tit had the greatest mean body length and the largest mean nest inner diameter, whereas the White‐crowned Penduline Tit population from HC had the smallest values for both traits.

Figure 5b shows species means (± SD) of beak size (PC1) and nest fiber width. The Black‐headed Penduline Tit had the highest mean PC1 score and the widest fibers, whereas the White‐crowned Penduline Tit had the lowest mean PC1 score and the narrowest fibers. The Chinese Penduline Tit was intermediate for both variables.

## 4 Discussion

In this study, we provide the first quantitative descriptions of nest structure for the White‐crowned Penduline Tit and Black‐headed Penduline Tit. These data add to the limited information currently available for the genus *Remiz* and provide a basis for comparative analyses of how morphology and ecological context may be associated with nest construction.

The genus *Remiz* comprises four species, including the three examined in this study and the Eurasian Penduline Tit. Taken together, previous studies and our results indicate that the Eurasian Penduline Tit constructs the tallest nests, with a mean height of 179.9 ± 2.5 mm (Szentirmai et al. 2005), whereas the other three species exhibit broadly similar nest heights (140.08 ± 11.91–151.43 ± 12.20 mm, Table 2). In contrast, inner diameter and entrance perimeter were greatest in the Black‐headed Penduline Tit, consistent with the possibility that the largest‐bodied species requires a larger nest entrance and a wider nest chamber.

Although the Black‐headed Penduline Tit exhibited the largest entrance perimeter, it constructed the shortest entrance corridor among the three species. In contrast, the Chinese and White‐crowned Penduline Tits exhibited substantially longer entrance tubes. The elongated entrance tube of penduline tit nests may be associated with both predator avoidance (Mainwaring et al. 2014; Martin et al. 2017). Tree‐nesting species occur in wooded environments where predation risk from snakes and small mammals is relatively high (Latif et al. 2012; Matysioková and Remeš 2024; Huang et al. 2025), whereas the Black‐headed Penduline Tit inhabits reed‐dominated wetlands, where overall predation pressure appears lower (Beauchamp 2023). Reduced predation risk may lessen the selective advantage of long corridors, resulting in shorter entrance structures (Vanadzina et al. 2023).

Despite the larger linear dimensions in Black‐headed Penduline Tit and its nest, the overall nest volume is relatively small. This pattern may be more closely related to differences in clutch size rather than to variation in nest insulation performance (Møller et al. 2014; Lee and Yoo 2016). Field observations indicate that the Black‐headed Penduline Tit lays 3–4 eggs per clutch (unpublished data), compared with 5–8 eggs in the Chinese Penduline Tit (Zheng et al. 2018), 5–9 eggs in the White‐crowned Penduline Tit, and 2–9 eggs in the Eurasian Penduline Tit (Valera et al. 1997; Czyż et al. 2024). Smaller clutch size may reduce the need for large internal nest volume, supporting the idea that nest volume may be associated with reproductive investment rather than body size alone (Soler 2001; Paillisson and Chambon 2021).

Nest thickness has been suggested to be associated with ambient temperature in the Eurasian Penduline Tit, with thicker nest walls enhancing thermal insulation and buffering thermal fluctuations during incubation (Szentirmai et al. 2005; Heenan 2013). In this study, we found that the Black‐headed Penduline Tit exhibited the thinnest nest bottoms and side walls among the three species examined. This pattern may be related to its breeding habitat. Unlike the tree‐nesting Chinese and White‐crowned Penduline Tits, the Black‐headed Penduline Tit constructs its nests within dense reed beds, where surrounding vegetation can provide substantial wind shelter and microclimatic buffering. Reed stands may reduce convective heat loss and dampen temperature fluctuations (Warrilow et al. 1978), thereby lowering the energetic demands associated with thermoregulation.

Nest material composition differed among species. The Chinese Penduline Tit primarily used shredded plant stems, plant down, and small amounts of wool. The White‐crowned Penduline Tit incorporated similar materials but in markedly different proportions, with wool comprising about 30% of total nest mass. Variation in nest materials may be associated with the availability of nesting materials in the local environment (Briggs and Deeming 2021; Sugasawa et al. 2025). At HC, where the White‐crowned Penduline Tit was studied, livestock grazing may increase the local availability of wool. The high wool content in this species may therefore reflect both local availability and the known insulating value of soft animal‐derived materials in bird nests (van Riper 1977; Surgey et al. 2012). The high wool content may enhance thermal insulation but also contributes to the lower overall nest mass observed in this species compared with the Chinese Penduline Tit (23.45 ± 5.60 g vs. 31.07 ± 6.65 g). However, mud located between the structural and lining layers may compensate for this reduced mass. Unlike in species such as the Barn Swallow ( *Hirundo rustica* ), where mud provides adhesive support (Deeming 2023), mud in the White‐crowned Penduline Tit may primarily increase nest mass, potentially reducing wind‐induced swaying and egg collisions. An important limitation of this study is that we did not quantify the availability of nesting materials at each study site. Consequently, differences in nest material composition among species and populations cannot be fully disentangled from differences in local material supply. Because species, site, and year were partially confounded in our sampling design, the observed patterns should not be interpreted as evidence of species‐specific material preferences. Future studies would benefit from simultaneously quantifying environmental material availability and nest material use, allowing stronger inference about the relative contributions of resource availability and species‐level selection in shaping nest construction.

Finally, our exploratory comparisons suggested that species with larger mean beak size tended to use wider nest fibers. The Black‐headed Penduline Tit, which has the largest beak among the three species examined, used the widest plant fibers in nest construction. Because the beak serves as the primary tool for grasping, cutting, and manipulating nesting materials, its size and shape may influence material selection (Sheard et al. 2023). Larger beaks may allow individuals to handle wider and possibly stiffer fibers more effectively.

In conclusion, we quantified nest structural traits and material composition across three penduline tit species, generating important comparative data for a genus celebrated for its highly specialized nest architecture. Our study provides the first quantitative descriptions of nest structure for the White‐crowned Penduline Tit and the Black‐headed Penduline Tit, addressing a notable gap in the natural history of this group. While the associations between morphological traits and nest characteristics reported here remain exploratory, our findings underscore the potential of penduline tits as a valuable study system for investigating how morphology, habitat, and material selection interact to shape nest construction. Together, these results call for more rigorous, experimentally grounded research into nest‐building behavior within a broader evolutionary and ecological framework.

## Author Contributions

**Zhe Hao:** data curation (lead), formal analysis (lead), investigation (equal), visualization (lead), writing – original draft (equal). **Yu Huang:** investigation (equal), methodology (equal), writing – original draft (equal). **Jiajun Jiang:** investigation (equal), methodology (equal), writing – original draft (equal). **Zhuoya Zhou:** investigation (equal), writing – original draft (supporting). **Wenni Jiang:** investigation (supporting), writing – original draft (supporting). **Bengao Tang:** investigation (supporting). **Kuerbanjiang Hanahati:** investigation (supporting). **Kedeerhan Bayaheng:** investigation (supporting). **Zhengwang Zhang:** funding acquisition (equal), resources (equal). **Hui Wang:** conceptualization (equal), funding acquisition (equal), writing – original draft (equal). **De Chen:** conceptualization (lead), funding acquisition (lead), investigation (supporting), project administration (lead), writing – review and editing (equal).

## Funding

This work was supported by National Natural Science Foundation of China, 31970405, 32161143024, 32500368. Amazing Biodiversity Discovery Small Grants Program.

## Conflicts of Interest

The authors declare no conflicts of interest.

## Data Availability

The nest structure data used in this study are publicly available at: https://github.com/Haozhe0316/A‐comparison‐of‐nest‐structure‐and‐materials‐in‐three‐species‐of‐penduline‐tits‐Remiz‐spp.‐/issues/1#issue‐4132347925.
